# Supplementary figures and images for: A familial Alzheimer’s disease associated mutation in presenilin-1 mediates amyloid-beta independent cell specific neurodegeneration
Source: PLoS One. 2024 Sep 6;19(9):e0289435. doi: 10.1371/journal.pone.0289435 (PMC11379242; doi:10.1371/journal.pone.0289435)

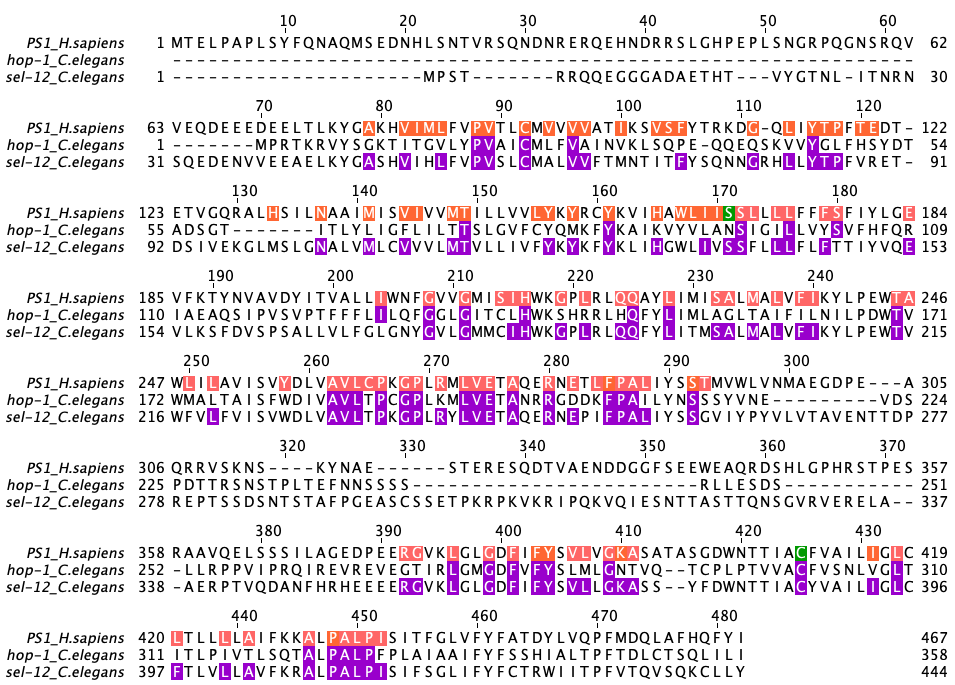

Supplement: S1 Fig — MUSCLE (Multiple Sequence Comparison by Log-Expectation) from the EMBL’s European Bioinformatics Institute Job Dispatcher (www.ebi.ac.uk/jdispatcher/msa) was used to generate multiple sequence alignment. Highlighted residues (red) are locations of known pathogenic or likely pathogenic variants of PS1. The two variants investigated in this study is highlighted in a different color (green). Amino acids that are identical between human PS1 and its C. elegans orthologs are or also highlighted (purple) in each respective sequence. (TIF) [file pone.0289435.s002.tif]

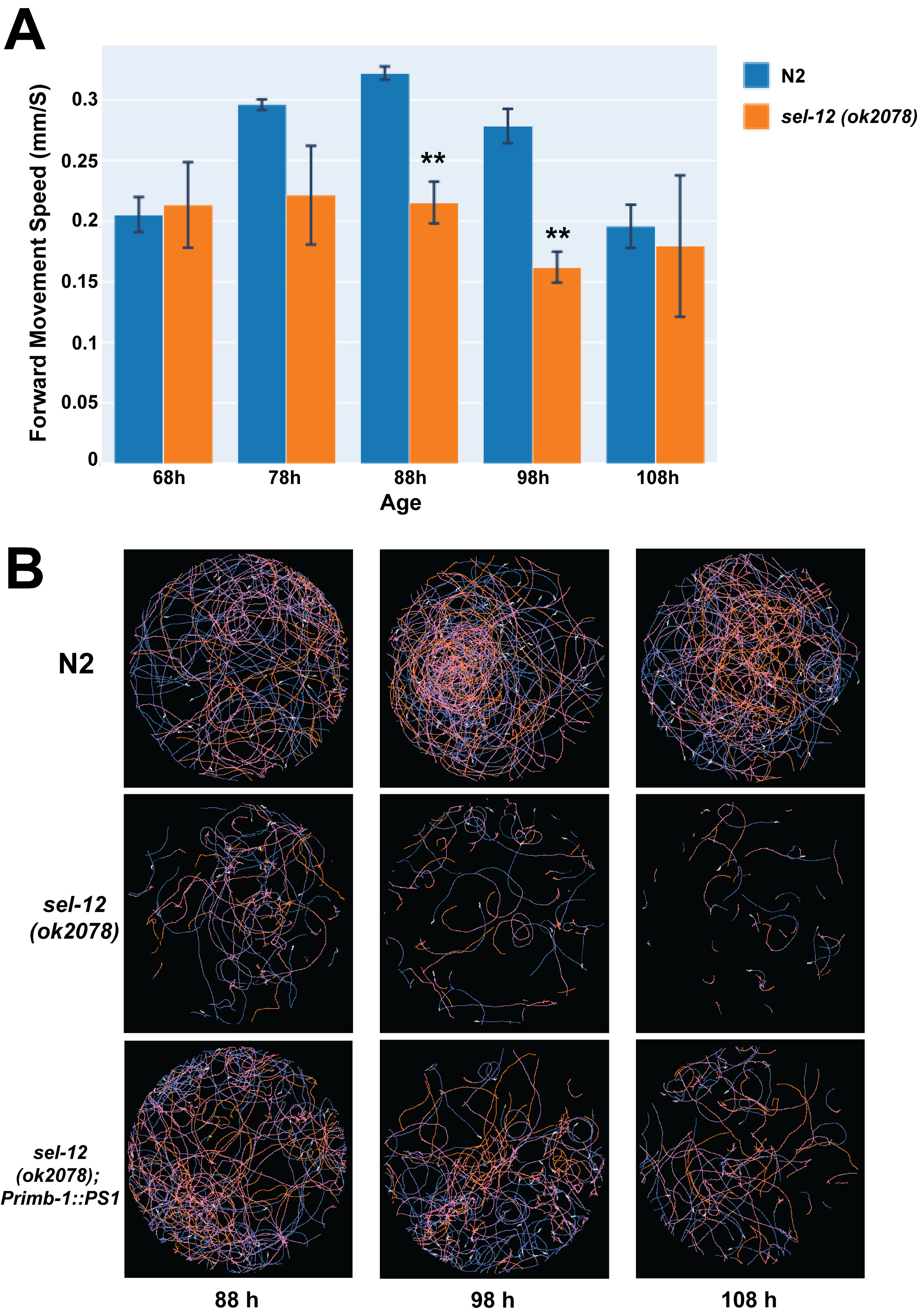

Supplement: S2 Fig — A) Average forward movement speed of 68–108 hour old wild-type and sel-12 mutant worms. Blue bars and red bars represent wild-type (N2) and sel-12 mutant worms, respectively. Each bar represents an average from 3 independent plates (n = 50 worms per plate). LOF: Loss of function. Error bars reflect the standard error of the mean. B) locomotion tracks of 88 to 108 hour old wild-type, sel-12 mutant, and nervous system PS1 rescue worms over a 250 second tracking period. Different colors indicate various tracking time points after a brief air-puff. (n = 50 worms per plate). One representative line is shown for the rescue experiment, as all three extrachromosomal lines followed the same trend. ** P<0.01. (TIF) [file pone.0289435.s003.tif]

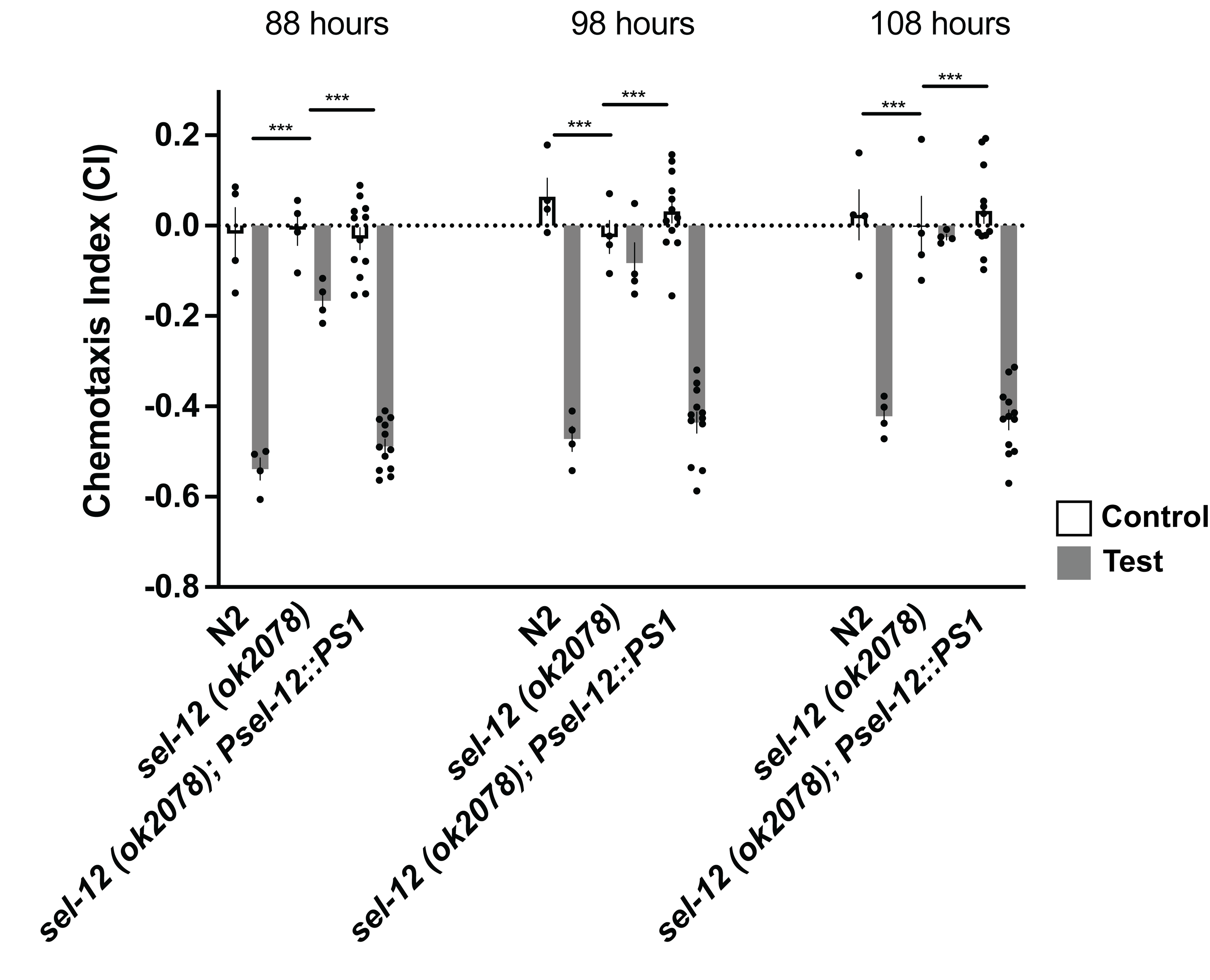

Supplement: S3 Fig — Octanol was used for test (aversive odorant) and M9 for controls (odorless buffer). Each bar represents an average of 4 independent plates (n = 50–100 worms per plate). Rescue bars are collapsed averages of 3 independent rescue lines generated. Independent plate CI are shown as circles. White bars represent control plates and black bars represent test plates. Error bars reflect the standard error of the mean.*** p<0.001. (TIF) [file pone.0289435.s004.tif]

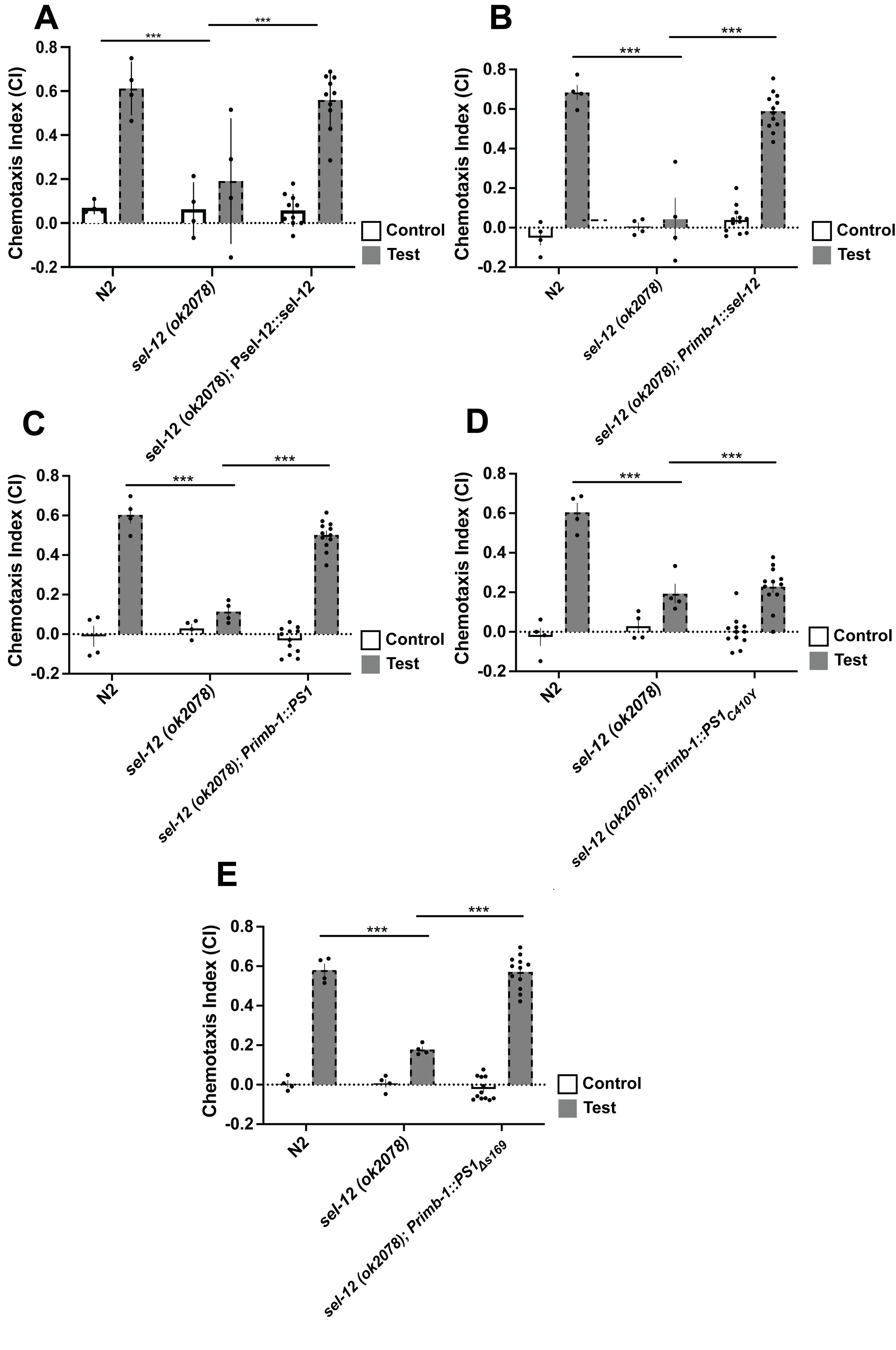

Supplement: S4 Fig — Psel-12::sel-12 (A), Primb-1::sel-12 (B), Primb-1::PS1 (C), and Primb-1::PS1Δs129 (E) rescued chemotaxis deficits on diacetyl, whereas Primb-1::PS1C410Y (D) did not. Diacetyl was used for test (attractive odorant) and M9 for control (odorless buffer). Independent plate CIs are shown as circles. Each bar represents and average from 4 independent plates (n = 50–100 synchronized 72 hr post egg-lay young adult worms per plate). Rescue bars are collapsed averages of 3 independent rescue lines generated. White bars represent control plates and black bars represent test plates. Error bars reflect the standard error of the mean. *** p<0.001. (TIF) [file pone.0289435.s005.tif]

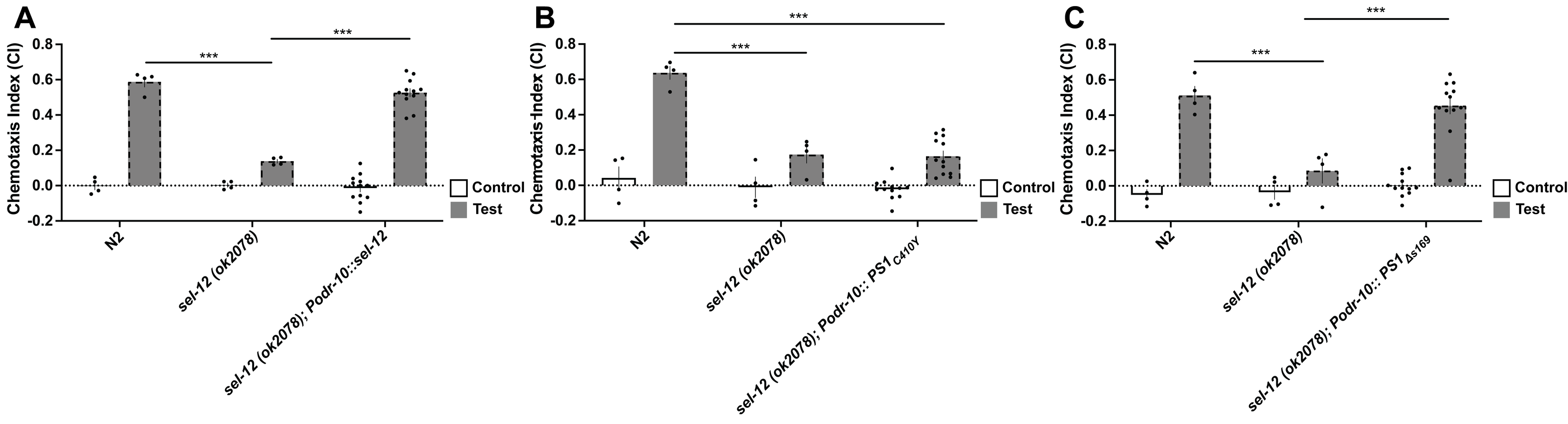

Supplement: S5 Fig — Podr-10::sel-12 (A) and Podr-10::PS1Δs169 (C) rescued chemotaxis deficits to diacetyl, whereas Podr-10::PS1C410Y (B) did not. Diacetyl was used for test (attractive odorant) and M9 for control (odorless buffer). Independent plate CI are shown as circles. Each bar represents an average from 4 independent plates (n = 50–100 synchronized 72 hr post egg-lay young adult worms per plate). Rescue bars are collapsed averages of 3 independent rescue lines generated. Whie bars represent control plates and black bars represent test plates. Error bars reflect the standard error of the mean. *** p<0.001. (TIF) [file pone.0289435.s006.tif]
